# Supplementary figures and images for: Immune-related pan-cancer gene expression signatures of patient survival revealed by NanoString-based analyses
Source: PLoS One. 2023 Jan 17;18(1):e0280364. doi: 10.1371/journal.pone.0280364 (PMC9844904; doi:10.1371/journal.pone.0280364)

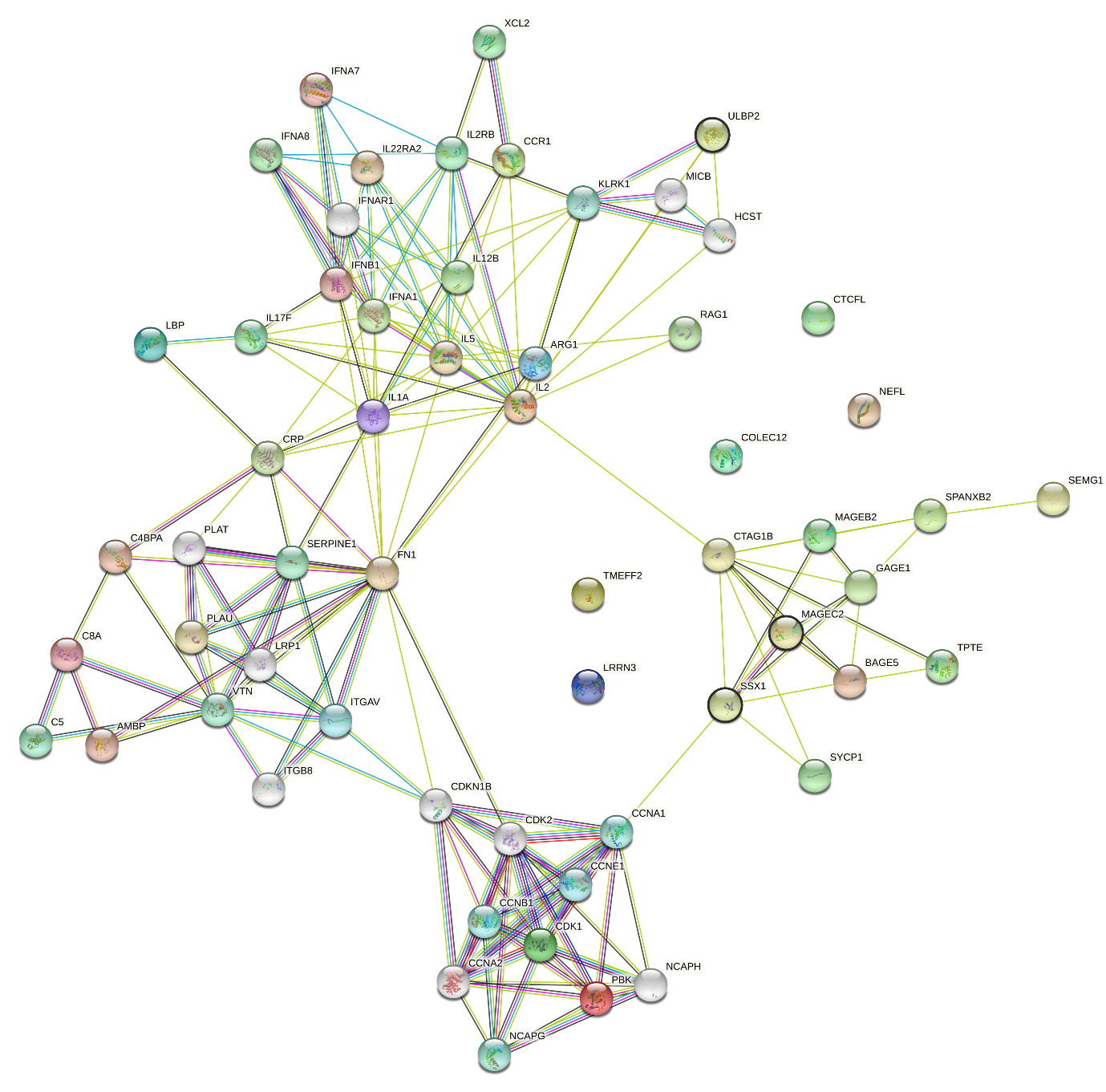

Supplement: S1 Fig — The panel shows expanded protein-protein interaction of up-regulated genes (n = 39) in short survival cancer patients (FDR < 0.05, LogFC > 0.5) within the whole cohort of cancer patients (n = 515). In bold those genes (SSX1, MAGEC2 and ULBP2) found shared across solid and blood patients. (TIF) [file pone.0280364.s001.tif]

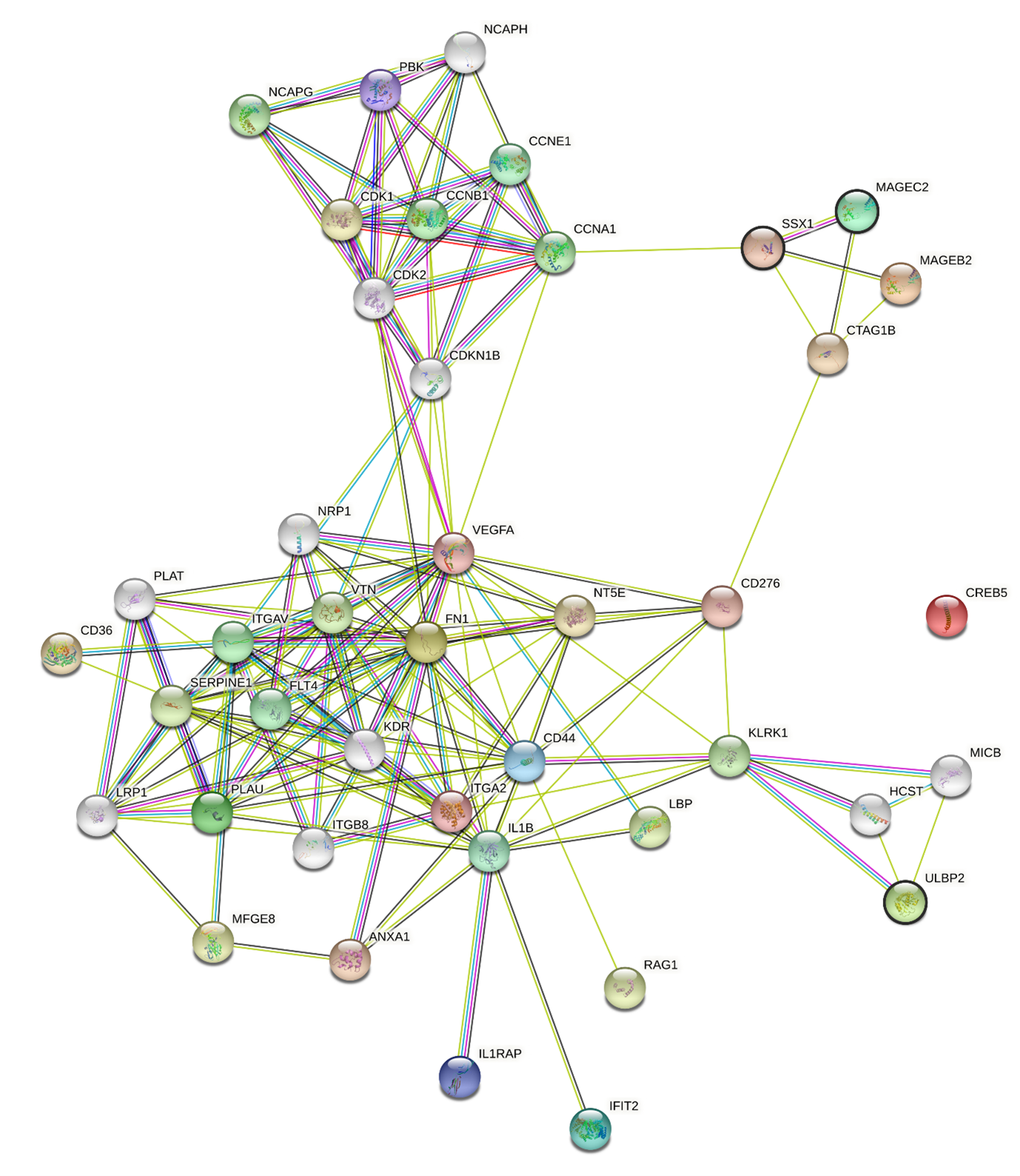

Supplement: S2 Fig — The panel shows the expanded network for genes found significantly up-regulated (Log FC greater and equal to 0.5) for solid cancer patients. In bold those genes (SSX1, MAGEC2 and ULBP2) found shared across solid and blood patients. (TIF) [file pone.0280364.s002.tif]

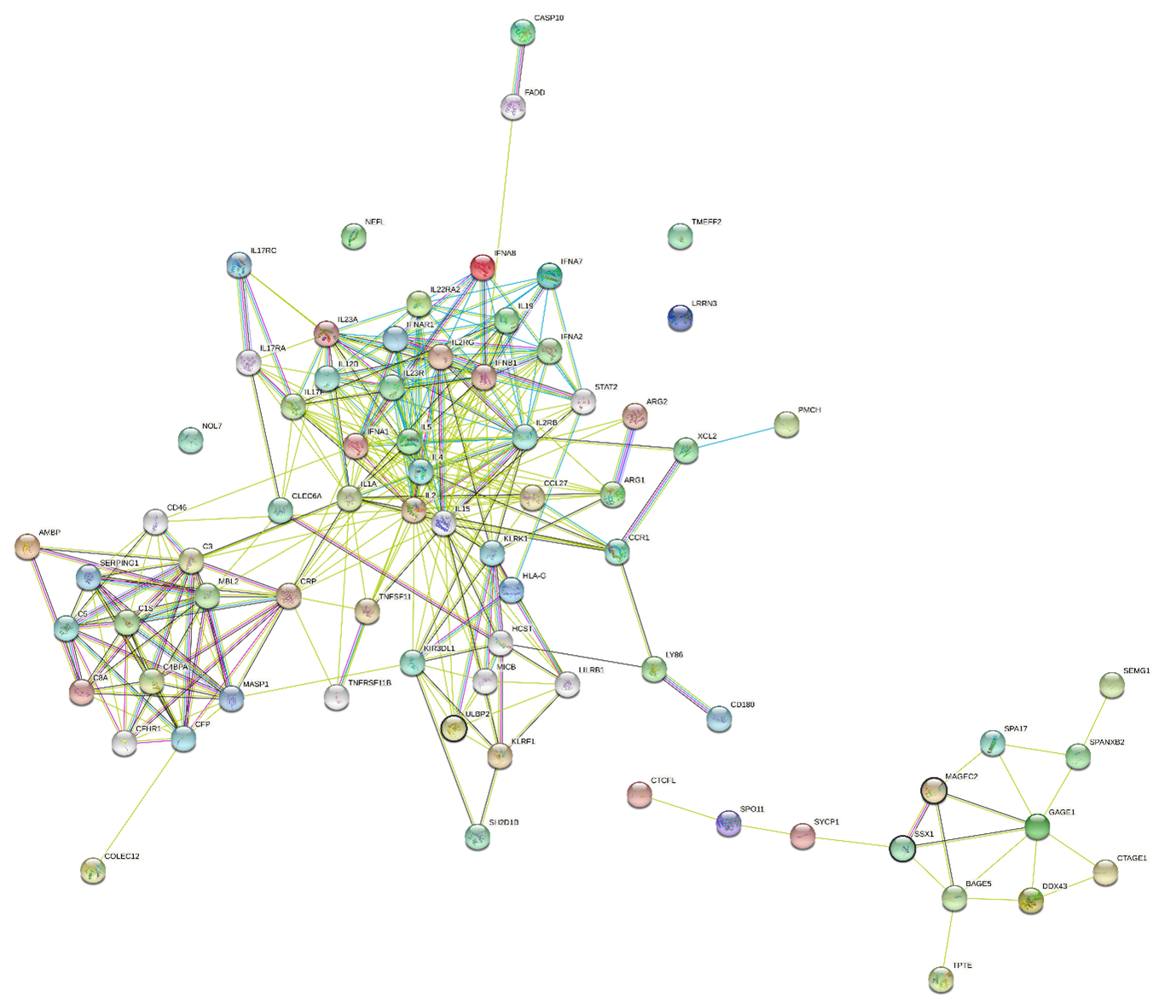

Supplement: S3 Fig — The panel reports the expanded network for genes found significantly up-regulated (Log FC greater and equal to 0.5) for blood patients. In bold those genes (SSX1, MAGEC2 and ULBP2) found shared across solid and blood patients. (TIF) [file pone.0280364.s003.tif]
