# Supplementary material for: Immune-related pan-cancer gene expression signatures of patient survival revealed by NanoString-based analyses
Source: PLoS One. 2023 Jan 17;18(1):e0280364. doi: 10.1371/journal.pone.0280364 (PMC9844904; doi:10.1371/journal.pone.0280364)
Supplement: S1 Table — (DOCX) [file pone.0280364.s004.docx]

Supplementary Table 1.

**Patients statistics**

| **Cancer types** | | | | | | | | | | | |
| --- | --- | --- | --- | --- | --- | --- | --- | --- | --- | --- | --- |
|  | | Solid | | | | | | | | Blood | |
|  | Total | Glioblastoma | Melanoma | Ovarian cancer | Head & Neck | Pancreatic cancer | Lung cancer | Colon cancer | Breast cancer | B Cell Lymphoma | Hodgkin Lymphoma |
| **Number of patients** | | | | | | | | | | | |
|  | 515 | 29 | 19 | 20 | 80 | 7 | 17 | 89 | 32 | 50 | 172 |
| **Percentage** | | | | | | | | | | | |
| Percentage | 100 | 56.9 | | | | | | | | 43.1 | |
|  | 100 | 5.63 | 3.69 | 3.9 | 15.53 | 1.36 | 3.30 | 17.28 | 6.21 | 9.7 | 33.40 |
| **Overall survival (months)** | | | | | | | | | | | |
| Survival range | 0.6-192 | 1.6-19.9 | 0.6-44 | 32-174 | 4-92 | 4-99 | 4-91 | 2-192 | 6-142 | 2-67 | 1-142 |
| Mean survival | 56.1 | 9.5 | 17.3 | 56.4 | 32.5 | 35.7 | 32.9 | 48.3 | 55.3 | 30.6 | 62.7 |
