# Supplementary material for: Immune-related pan-cancer gene expression signatures of patient survival revealed by NanoString-based analyses
Source: PLoS One. 2023 Jan 17;18(1):e0280364. doi: 10.1371/journal.pone.0280364 (PMC9844904; doi:10.1371/journal.pone.0280364)
Supplement: S2 Table — (DOCX) [file pone.0280364.s005.docx]

Supplementary Table 2

**Cohort description and gene expression dataset numbers**

|  | Cancer type | Number of patients | GSE number |
| --- | --- | --- | --- |
| 1 | Glioblastoma | 29 | Private |
| 2 | Melanoma | 19 | GSE124574 |
| 3 | Ovarian | 20 | EGAS00001002839 |
| 4 | Head&Neck | 80 | 1. GSE122272  2. Private |
| 5 | Pancreatic | 7 | GSE132946 |
| 6 | Lung | 17 | GSE161116 |
| 7 | Colon | 89 | Private |
| 8 | Breast | 32 | GSE102818 |
| 9 | Large B cell Lymphoma | 50 | GSE147115  GSE147116 |
| 10 | Hodgkin Lymphoma | 172 | Private |
