# Supplementary material for: Immune-related pan-cancer gene expression signatures of patient survival revealed by NanoString-based analyses
Source: PLoS One. 2023 Jan 17;18(1):e0280364. doi: 10.1371/journal.pone.0280364 (PMC9844904; doi:10.1371/journal.pone.0280364)
Supplement: S3 Table — (DOCX) [file pone.0280364.s006.docx]

Supplementary Table 3

**List of genes found upregulated in short survival patients among the whole cohort of patients**

| Gene symbol | Gene description | logFC | P value | FDR |
| --- | --- | --- | --- | --- |
| AMBP | alpha-1-microglobulin/bikunin precursor | 0.535952 | 0.00491 | 0.029042 |
| ARG1 | arginase 1 | 0.643185 | 0.003275 | 0.021903 |
| BAGE | B melanoma antigen | 0.501949 | 0.003622 | 0.023601 |
| C4BPA | complement component 4 binding protein alpha | 0.564754 | 0.001906 | 0.01451 |
| C8A | complement C8 alpha chain | 0.575178 | 0.002029 | 0.0153 |
| CCR1 | C-C motif chemokine receptor 1 | 0.522354 | 3.15E-07 | 6.06E-05 |
| CDK1 | cyclin dependent kinase 1 | 0.533706 | 5.39E-06 | 0.000207 |
| COLEC12 | collectin subfamily member 12 | 0.535526 | 1.02E-05 | 0.000356 |
| CRP | C-reactive protein | 0.560024 | 0.006102 | 0.033282 |
| CTAG1B | cancer/testis antigen 1B | 0.555629 | 0.000591 | 0.00693 |
| CTCFL | CCCTC-binding factor like | 0.983406 | 2.13E-05 | 0.000584 |
| FN1 | fibronectin 1 | 0.625033 | 0.001239 | 0.010359 |
| GAGE1 | G antigen 1 | 0.741072 | 1.12E-05 | 0.000358 |
| IFNA1 | interferon alpha 1 | 0.560617 | 0.010227 | 0.048847 |
| IFNA7 | interferon alpha 7 | 0.728127 | 0.003788 | 0.024274 |
| IFNA8 | interferon alpha 8 | 0.721209 | 0.002074 | 0.015338 |
| IFNB1 | interferon beta 1 | 0.570879 | 0.004612 | 0.027705 |
| IL12B | interleukin 12B | 0.608256 | 0.000608 | 0.00693 |
| IL17F | interleukin 17F | 0.536286 | 0.005374 | 0.030609 |
| IL1A | interleukin 1 alpha | 0.595399 | 0.001873 | 0.01451 |
| IL2 | interleukin 2 | 0.536878 | 0.003566 | 0.023439 |
| IL22RA2 | interleukin 22 receptor subunit alpha 2 | 0.580837 | 0.000293 | 0.004598 |
| IL5 | interleukin 5 | 0.809436 | 5.13E-05 | 0.001195 |
| LBP | lipopolysaccharide binding protein | 0.541858 | 0.002251 | 0.016329 |
| LRRN3 | leucine rich repeat neuronal 3 | 0.564614 | 0.000869 | 0.008352 |
| MAGEB2 | MAGE family member B2 | 0.720639 | 0.000326 | 0.004961 |
| MAGEC2 | MAGE family member C2 | 1.029034 | 3.85E-06 | 0.000183 |
| NEFL | neurofilament light | 0.593523 | 0.006922 | 0.03579 |
| PBK | PDZ binding kinase | 0.564125 | 6.12E-05 | 0.001384 |
| PLAU | plasminogen activator urokinase | 0.63534 | 2.77E-06 | 0.000177 |
| RAG1 | recombination activating 1 | 0.641975 | 9.19E-05 | 0.001909 |
| SEMG1 | semenogelin 1 | 0.515549 | 0.003864 | 0.024559 |
| SPANXB1 | SPANX family member B1 | 0.932128 | 1.84E-05 | 0.000546 |
| SSX1 | SSX family member 1 | 1.162607 | 2.05E-08 | 1.58E-05 |
| SYCP1 | synaptonemal complex protein 1 | 0.533364 | 0.001183 | 0.01011 |
| TMEFF2 | transmembrane protein with EGF like and two follistatin like domains 2 | 0.631505 | 0.000335 | 0.004961 |
| TPTE | transmembrane phosphatase with tensin homology | 0.691593 | 0.000331 | 0.004961 |
| ULBP2 | UL16 binding protein 2 | 0.975494 | 3.54E-06 | 0.000183 |
| XCL2 | X-C motif chemokine ligand 2 | 0.636899 | 2.12E-05 | 0.000584 |
