# Supplementary material for: Immune-related pan-cancer gene expression signatures of patient survival revealed by NanoString-based analyses
Source: PLoS One. 2023 Jan 17;18(1):e0280364. doi: 10.1371/journal.pone.0280364 (PMC9844904; doi:10.1371/journal.pone.0280364)
Supplement: S4 Table — (DOCX) [file pone.0280364.s007.docx]

Supplementary Table 4

**List of genes found upregulated in short survival patients among solid patients**

| Gene symbol | logFC | logCPM | F | PValue | FDR | Gene.description |
| --- | --- | --- | --- | --- | --- | --- |
| ANXA1 | 0.62441 | 14.41303 | 14.274 | 0.000192 | 0.007907 | annexin A1 [Source:HGNC Symbol;Acc:HGNC:533] |
| CD276 | 0.543015 | 11.27916 | 23.53097 | 2.01E-06 | 0.000221 | CD276 molecule [Source:HGNC Symbol;Acc:HGNC:19137] |
| CD36 | 0.822982 | 9.629789 | 19.00043 | 1.82E-05 | 0.001077 | CD36 molecule [Source:HGNC Symbol;Acc:HGNC:1663] |
| CD44 | 0.504513 | 13.64323 | 9.539335 | 0.002207 | 0.03683 | CD44 molecule (Indian blood group) [Source:HGNC Symbol;Acc:HGNC:1681] |
| CDK1 | 0.621264 | 10.14293 | 14.18378 | 0.000201 | 0.007907 | cyclin dependent kinase 1 [Source:HGNC Symbol;Acc:HGNC:1722] |
| CREB5 | 0.536546 | 8.56241 | 15.12958 | 0.000125 | 0.00599 | cAMP responsive element binding protein 5 [Source:HGNC Symbol;Acc:HGNC:16844] |
| CTAG1B | 1.024268 | 6.775933 | 13.16209 | 0.000338 | 0.010392 | cancer/testis antigen 1B [Source:HGNC Symbol;Acc:HGNC:2491] |
| FN1 | 0.71756 | 13.50004 | 10.48015 | 0.001347 | 0.029588 | fibronectin 1 [Source:HGNC Symbol;Acc:HGNC:3778] |
| IFIT2 | 0.574657 | 8.747354 | 13.04588 | 0.000358 | 0.010598 | interferon induced protein with tetratricopeptide repeats 2 [Source:HGNC Symbol;Acc:HGNC:5409] |
| IL1RAP | 0.664554 | 9.779418 | 25.10196 | 9.49E-07 | 0.000146 | interleukin 1 receptor accessory protein [Source:HGNC Symbol;Acc:HGNC:5995] |
| ITGA2 | 0.509289 | 9.881646 | 12.63839 | 0.000441 | 0.011313 | integrin subunit alpha 2 [Source:HGNC Symbol;Acc:HGNC:6137] |
| LBP | 0.639185 | 5.754546 | 8.827872 | 0.003216 | 0.04756 | lipopolysaccharide binding protein [Source:HGNC Symbol;Acc:HGNC:6517] |
| MAGEB2 | 1.370987 | 5.944315 | 24.6817 | 1.16E-06 | 0.000149 | MAGE family member B2 [Source:HGNC Symbol;Acc:HGNC:6809] |
| MAGEC2 | 1.071639 | 6.157865 | 13.17077 | 0.000336 | 0.010392 | MAGE family member C2 [Source:HGNC Symbol;Acc:HGNC:13574] |
| MFGE8 | 0.511373 | 11.4892 | 13.41767 | 0.000296 | 0.010325 | milk fat globule EGF and factor V/VIII domain containing [Source:HGNC Symbol;Acc:HGNC:7036] |
| NT5E | 0.538531 | 9.940836 | 9.821179 | 0.001903 | 0.033847 | 5'-nucleotidase ecto [Source:HGNC Symbol;Acc:HGNC:8021] |
| PBK | 0.658045 | 8.535169 | 11.62792 | 0.000742 | 0.017838 | PDZ binding kinase [Source:HGNC Symbol;Acc:HGNC:18282] |
| PLAU | 1.023943 | 11.36538 | 29.15187 | 1.40E-07 | 3.58E-05 | plasminogen activator, urokinase [Source:HGNC Symbol;Acc:HGNC:9052] |
| RAG1 | 0.605631 | 5.654563 | 15.82643 | 8.78E-05 | 0.004503 | recombination activating 1 [Source:HGNC Symbol;Acc:HGNC:9831] |
| SSX1 | 1.472692 | 5.48099 | 31.82148 | 4.03E-08 | 1.55E-05 | SSX family member 1 [Source:HGNC Symbol;Acc:HGNC:11335] |
| ULBP2 | 0.823042 | 7.255068 | 21.20195 | 6.20E-06 | 0.000477 | UL16 binding protein 2 [Source:HGNC Symbol;Acc:HGNC:14894] |
| VEGFA | 0.642779 | 11.96688 | 12.86026 | 0.000394 | 0.011072 | vascular endothelial growth factor A [Source:HGNC Symbol;Acc:HGNC:12680] |
