# Supplementary material for: Immune-related pan-cancer gene expression signatures of patient survival revealed by NanoString-based analyses
Source: PLoS One. 2023 Jan 17;18(1):e0280364. doi: 10.1371/journal.pone.0280364 (PMC9844904; doi:10.1371/journal.pone.0280364)
Supplement: S5 Table — (DOCX) [file pone.0280364.s008.docx]

Supplementary Table 5

**List of genes found upregulated in short survival patients among blood patients.**

| Gene symbol | logFC | logCPM | F | PValue | FDR | Gene.description |
| --- | --- | --- | --- | --- | --- | --- |
| AMBP | 1.209309 | 5.764609 | 13.18668 | 0.00035 | 0.006117 | alpha-1-microglobulin/bikunin precursor [Source:HGNC Symbol;Acc:HGNC:453] |
| ARG1 | 1.239715 | 5.778596 | 11.11169 | 0.001005 | 0.011039 | arginase 1 [Source:HGNC Symbol;Acc:HGNC:663] |
| ARG2 | 0.678679 | 5.477748 | 12.11658 | 0.000601 | 0.008304 | arginase 2 [Source:HGNC Symbol;Acc:HGNC:664] |
| BAGE | 0.937339 | 5.730471 | 10.02935 | 0.001757 | 0.015709 | B melanoma antigen |
| C1S | 0.581397 | 10.88253 | 14.11945 | 0.000219 | 0.004605 | complement C1s [Source:HGNC Symbol;Acc:HGNC:1247] |
| C3 | 0.915878 | 10.85067 | 19.41434 | 1.64E-05 | 0.00074 | complement C3 [Source:HGNC Symbol;Acc:HGNC:1318] |
| C4BPA | 0.875111 | 5.098353 | 8.125773 | 0.004775 | 0.032179 | complement component 4 binding protein alpha [Source:HGNC Symbol;Acc:HGNC:1325] |
| C8A | 1.098138 | 5.39071 | 10.56599 | 0.001331 | 0.013198 | complement C8 alpha chain [Source:HGNC Symbol;Acc:HGNC:1352] |
| CASP10 | 0.844053 | 6.122655 | 8.191397 | 0.004611 | 0.031845 | caspase 10 [Source:HGNC Symbol;Acc:HGNC:1500] |
| CCL27 | 0.787697 | 4.730366 | 10.55431 | 0.001339 | 0.013198 | C-C motif chemokine ligand 27 [Source:HGNC Symbol;Acc:HGNC:10626] |
| CCR1 | 0.697434 | 7.623503 | 14.66997 | 0.000167 | 0.004004 | C-C motif chemokine receptor 1 [Source:HGNC Symbol;Acc:HGNC:1602] |
| CLEC6A | 0.633897 | 6.46941 | 13.64996 | 0.000277 | 0.0052 | C-type lectin domain containing 6A [Source:HGNC Symbol;Acc:HGNC:14556] |
| COLEC12 | 0.972663 | 6.672579 | 29.733 | 1.31E-07 | 2.53E-05 | collectin subfamily member 12 [Source:HGNC Symbol;Acc:HGNC:16016] |
| CRP | 1.095183 | 5.275923 | 8.670261 | 0.003578 | 0.026457 | C-reactive protein [Source:HGNC Symbol;Acc:HGNC:2367] |
| CTAGE1 | 0.913424 | 5.643648 | 7.152081 | 0.008044 | 0.045151 | cutaneous T cell lymphoma-associated antigen 1 [Source:HGNC Symbol;Acc:HGNC:24346] |
| CTCFL | 1.394481 | 6.131318 | 12.9653 | 0.000391 | 0.006542 | CCCTC-binding factor like [Source:HGNC Symbol;Acc:HGNC:16234] |
| DDX43 | 0.718873 | 5.740651 | 8.004352 | 0.005093 | 0.033193 | DEAD-box helicase 43 [Source:HGNC Symbol;Acc:HGNC:18677] |
| GAGE1 | 1.527198 | 5.161384 | 30.55219 | 9.07E-08 | 2.32E-05 | G antigen 1 [Source:HGNC Symbol;Acc:HGNC:4098] |
| HLA-G | 0.649275 | 10.56519 | 15.06591 | 0.000137 | 0.004004 | major histocompatibility complex, class I, G [Source:HGNC Symbol;Acc:HGNC:4964] |
| IFNA1 | 1.205476 | 5.823644 | 9.398747 | 0.00244 | 0.020397 | interferon alpha 1 [Source:HGNC Symbol;Acc:HGNC:5417] |
| IFNA2 | 1.143504 | 5.874173 | 10.51988 | 0.001363 | 0.013265 | interferon alpha 2 [Source:HGNC Symbol;Acc:HGNC:5423] |
| IFNA7 | 1.386182 | 7.965624 | 8.877956 | 0.003207 | 0.025165 | interferon alpha 7 [Source:HGNC Symbol;Acc:HGNC:5428] |
| IFNA8 | 1.395609 | 5.797207 | 10.30121 | 0.001526 | 0.014433 | interferon alpha 8 [Source:HGNC Symbol;Acc:HGNC:5429] |
| IFNB1 | 1.108403 | 5.958149 | 8.621916 | 0.003671 | 0.026883 | interferon beta 1 [Source:HGNC Symbol;Acc:HGNC:5434] |
| IL12B | 1.259474 | 5.695796 | 14.8372 | 0.000153 | 0.004004 | interleukin 12B [Source:HGNC Symbol;Acc:HGNC:5970] |
| IL17F | 1.149857 | 6.012723 | 10.15353 | 0.001647 | 0.014902 | interleukin 17F [Source:HGNC Symbol;Acc:HGNC:16404] |
| IL19 | 0.546571 | 4.392174 | 7.887057 | 0.005422 | 0.033984 | interleukin 19 [Source:HGNC Symbol;Acc:HGNC:5990] |
| IL1A | 1.322529 | 5.932225 | 15.42525 | 0.000115 | 0.003524 | interleukin 1 alpha [Source:HGNC Symbol;Acc:HGNC:5991] |
| IL2 | 1.140629 | 5.6595 | 11.91252 | 0.000667 | 0.008561 | interleukin 2 [Source:HGNC Symbol;Acc:HGNC:6001] |
| IL22RA2 | 1.037536 | 7.755083 | 14.21497 | 0.000209 | 0.004605 | interleukin 22 receptor subunit alpha 2 [Source:HGNC Symbol;Acc:HGNC:14901] |
| IL23A | 0.514414 | 6.195252 | 6.829842 | 0.009577 | 0.049428 | interleukin 23 subunit alpha [Source:HGNC Symbol;Acc:HGNC:15488] |
| IL23R | 0.751974 | 5.69783 | 6.874293 | 0.009349 | 0.04924 | interleukin 23 receptor [Source:HGNC Symbol;Acc:HGNC:19100] |
| IL2RG | 0.534399 | 11.14993 | 14.44841 | 0.000186 | 0.004335 | interleukin 2 receptor subunit gamma [Source:HGNC Symbol;Acc:HGNC:6010] |
| IL5 | 1.454116 | 5.510716 | 15.9346 | 8.91E-05 | 0.002854 | interleukin 5 [Source:HGNC Symbol;Acc:HGNC:6016] |
| KIR3DL1 | 0.813692 | 5.301099 | 7.913476 | 0.005346 | 0.033984 | killer cell immunoglobulin like receptor, three Ig domains and long cytoplasmic tail 1 [Source:HGNC Symbol;Acc:HGNC:6338] |
| KLRF1 | 0.74268 | 6.224018 | 12.57435 | 0.000477 | 0.007483 | killer cell lectin like receptor F1 [Source:HGNC Symbol;Acc:HGNC:13342] |
| LRRN3 | 1.115945 | 6.218807 | 13.96063 | 0.000237 | 0.00468 | leucine rich repeat neuronal 3 [Source:HGNC Symbol;Acc:HGNC:17200] |
| LY86 | 0.655934 | 8.291418 | 22.5518 | 3.67E-06 | 0.000256 | lymphocyte antigen 86 [Source:HGNC Symbol;Acc:HGNC:16837] |
| MAGEC2 | 1.013335 | 5.561338 | 8.929539 | 0.003121 | 0.024744 | MAGE family member C2 [Source:HGNC Symbol;Acc:HGNC:13574] |
| MBL2 | 0.67139 | 4.540787 | 8.823216 | 0.003301 | 0.025639 | mannose binding lectin 2 [Source:HGNC Symbol;Acc:HGNC:6922] |
| NEFL | 1.181511 | 5.527123 | 12.44368 | 0.000509 | 0.007681 | neurofilament light [Source:HGNC Symbol;Acc:HGNC:7739] |
| NOL7 | 0.651762 | 8.905213 | 20.56144 | 9.44E-06 | 0.000484 | nucleolar protein 7 [Source:HGNC Symbol;Acc:HGNC:21040] |
| PMCH | 0.905208 | 6.235816 | 8.673961 | 0.003571 | 0.026457 | pro-melanin concentrating hormone [Source:HGNC Symbol;Acc:HGNC:9109] |
| SEMG1 | 1.17375 | 5.418398 | 14.89377 | 0.000149 | 0.004004 | semenogelin 1 [Source:HGNC Symbol;Acc:HGNC:10742] |
| SH2D1B | 0.679992 | 6.147205 | 8.585738 | 0.003741 | 0.027143 | SH2 domain containing 1B [Source:HGNC Symbol;Acc:HGNC:30416] |
| SPA17 | 0.568298 | 7.258853 | 8.968287 | 0.003058 | 0.024744 | sperm autoantigenic protein 17 [Source:HGNC Symbol;Acc:HGNC:11210] |
| SPANXB1 | 1.471642 | 5.45489 | 14.80831 | 0.000156 | 0.004004 | SPANX family member B1 [Source:HGNC Symbol;Acc:HGNC:14329] |
| SPO11 | 1.008023 | 5.174174 | 9.10072 | 0.002853 | 0.023588 | SPO11 initiator of meiotic double stranded breaks [Source:HGNC Symbol;Acc:HGNC:11250] |
| SSX1 | 0.891981 | 5.864232 | 8.0947 | 0.004854 | 0.032179 | SSX family member 1 [Source:HGNC Symbol;Acc:HGNC:11335] |
| SYCP1 | 1.060709 | 5.524426 | 13.37318 | 0.000319 | 0.005835 | synaptonemal complex protein 1 [Source:HGNC Symbol;Acc:HGNC:11487] |
| TMEFF2 | 0.943194 | 5.568702 | 10.64178 | 0.00128 | 0.012948 | transmembrane protein with EGF like and two follistatin like domains 2 [Source:HGNC Symbol;Acc:HGNC:11867] |
| TNFSF11 | 0.585225 | 6.04941 | 11.81339 | 0.000702 | 0.008565 | TNF superfamily member 11 [Source:HGNC Symbol;Acc:HGNC:11926] |
| TPTE | 1.3437 | 6.386418 | 14.09483 | 0.000222 | 0.004605 | transmembrane phosphatase with tensin homology [Source:HGNC Symbol;Acc:HGNC:12023] |
| ULBP2 | 1.17007 | 6.742651 | 8.096846 | 0.004849 | 0.032179 | UL16 binding protein 2 [Source:HGNC Symbol;Acc:HGNC:14894] |
| XCL2 | 0.931021 | 7.213217 | 11.84591 | 0.00069 | 0.008561 | X-C motif chemokine ligand 2 [Source:HGNC Symbol;Acc:HGNC:10646] |
