# Supplementary material for: Immune-related pan-cancer gene expression signatures of patient survival revealed by NanoString-based analyses
Source: PLoS One. 2023 Jan 17;18(1):e0280364. doi: 10.1371/journal.pone.0280364 (PMC9844904; doi:10.1371/journal.pone.0280364)
Supplement: S6 Table — (DOCX) [file pone.0280364.s009.docx]

Supplementary Table 6

**CIBERSORT tables reporting values of 22 types of immune cell proportions for all patients, solid and blood cancer patients respectively in a long versus short survival comparison.**

| Immune cell type | All patients | | Solid cancer patients | | Blood cancer patients | |
| --- | --- | --- | --- | --- | --- | --- |
|  | Long OS | Short OS | Long OS | Short OS | Long OS | Short OS |
| B cells naive | 0.117 | 0.097 | 0.099 | 0.089 | 0.140 | 0.107 |
| B cells memory | 0.053 | 0.065 | 0.012 | 0.007 | 0.106 | 0.137 |
| Plasma cells | 0.048 | 0.041 | 0.045 | 0.042 | 0.052 | 0.040 |
| T cells CD8 | 0.049 | 0.047 | 0.042 | 0.043 | 0.058 | 0.053 |
| T cells CD4 naive | 0.024 | 0.030 | 0.013 | 0.021 | 0.038 | 0.041 |
| T cells CD4 memory resting | 0.094 | 0.075 | 0.109 | 0.074 | 0.074 | 0.078 |
| T cells CD4 memory activated | 0.0166 | 0.020 | 0.011 | 0.014 | 0.023 | 0.028 |
| T cells follicular helper | 0.039 | 0.044 | 0.021 | 0.019 | 0.062 | 0.075 |
| T cells regulatory Tregs | 0.031 | 0.020 | 0.019 | 0.014 | 0.046 | 0.027 |
| T cells gamma delta | 0.026 | 0.032 | 0.019 | 0.023 | 0.034 | 0.044 |
| NK cells resting | 0.013 | 0.014 | 0.017 | 0.021 | 0.008 | 0.006 |
| NK cells activated | 0.041 | 0.039 | 0.047 | 0.044 | 0.033 | 0.032 |
| Monocytes | 0.008 | 0.013 | 0.012 | 0.023 | 0.003 | 0.001 |
| Macrophages M0 | 0.102 | 0.106 | 0.142 | 0.146 | 0.051 | 0.055 |
| Macrophages M1 | 0.078 | 0.078 | 0.078 | 0.082 | 0.079 | 0.073 |
| Macrophages M2 | 0.119 | 0.140 | 0.123 | 0.151 | 0.115 | 0.125 |
| Dendritic cells resting | 0.002 | 0.003 | 0.004 | 0.004 | 0.0003 | 0.001 |
| Dendritic cells activated | 0.006 | 0.008 | 0.005 | 0.006 | 0.007 | 0.010 |
| Mast cells resting | 0.071 | 0.064 | 0.092 | 0.078 | 0.044 | 0.045 |
| Mast cells activated | 0.017 | 0.016 | 0.028 | 0.028 | 0.002 | 0.0008 |
| Eosinophils | 0.008 | 0.009 | 0.013 | 0.016 | 0.002 | 0.001 |
| Neutrophils | 0.027 | 0.029 | 0.037 | 0.043 | 0.013 | 0.010 |
| Abbreviations: NK, natural killer; OS, overall survival | | | | | | |
