# Supplementary material for: Immune-related pan-cancer gene expression signatures of patient survival revealed by NanoString-based analyses
Source: PLoS One. 2023 Jan 17;18(1):e0280364. doi: 10.1371/journal.pone.0280364 (PMC9844904; doi:10.1371/journal.pone.0280364)
Supplement: S7 Table — (DOCX) [file pone.0280364.s010.docx]

Supplementary Table 7

**CIBERSORT table reporting values of 22 types of immune cell proportions according to cancer type.**

|  | Breast Cancer | Colon Cancer | Glioblastoma | Lung Cancer | Melanoma | Ovarian Cancer | Pancreas Cancer | Head and Neck Cancer | B Cell Lymphoma | Hodgkin Lymphoma |
| --- | --- | --- | --- | --- | --- | --- | --- | --- | --- | --- |
| B cells naive | 2.674751 | 8.345049 | 1.867032 | 1.131624 | 1.017837 | 1.735059 | 1.293472 | 8.888656 | 8.211454 | 19.01445 |
| B cells memory | 0.420676 | 0.262917 | 0 | 0.461569 | 0.919089 | 0.064882 | 0 | 0.890588 | 11.25989 | 14.57744 |
| Plasma cells | 0.77671 | 3.381007 | 1.189906 | 0.650386 | 0.764017 | 1.010445 | 0.027173 | 5.076037 | 3.288348 | 6.961714 |
| T cells CD8 | 1.462163 | 4.495111 | 0.673856 | 0.964713 | 0.480978 | 0.793223 | 0.471889 | 2.730312 | 2.326954 | 9.401943 |
| T cells CD4 naive | 1.16935 | 1.1255 | 0.498525 | 0.00626 | 0.827413 | 0.295173 | 0.009493 | 1.455493 | 2.15874 | 7.431915 |
| T cells CD4 memory resting | 2.789203 | 8.920099 | 0.368962 | 3.532105 | 1.057987 | 1.925266 | 0.310864 | 7.529518 | 1.42864 | 16.52205 |
| T cells CD4 memory activated | 0.091278 | 1.721088 | 0.107381 | 0 | 0.294338 | 0.131407 | 0 | 1.029954 | 1.148814 | 4.364415 |
| T cells follicular helper | 0.274499 | 2.223139 | 0.451192 | 0.116943 | 0.399308 | 0.419482 | 0.168781 | 1.073958 | 1.389824 | 13.93059 |
| T cells regulatory Tregs | 0.466768 | 0.817702 | 0.075148 | 0.286868 | 0.017988 | 0.419848 | 0.453402 | 2.197179 | 0.348781 | 6.910011 |
| T cells gamma delta | 0.425557 | 2.22758 | 1.146834 | 0.294586 | 1.525414 | 0.550207 | 0.04564 | 0.162446 | 2.182841 | 6.08806 |
| NK cells resting | 0.16086 | 1.514808 | 0.13033 | 0.025717 | 0.472046 | 0.126974 | 0 | 3.420181 | 0.350062 | 1.339316 |
| NK cells activated | 1.704803 | 4.326926 | 1.125814 | 1.173287 | 0.675381 | 1.302331 | 0.494836 | 2.057719 | 1.774121 | 5.43142 |
| Monocytes | 0.422527 | 0.210061 | 2.53735 | 0.079041 | 0.613593 | 0.604279 | 0.102468 | 0.70872 | 0.087316 | 0.532168 |
| Macrophages M0 | 3.15453 | 16.3444 | 5.098487 | 1.744089 | 1.429942 | 1.926823 | 1.332803 | 12.98664 | 4.257826 | 9.027826 |
| Macrophages M1 | 2.650287 | 7.805026 | 0.911052 | 0.911241 | 1.022383 | 2.566718 | 0.176249 | 6.237913 | 3.115592 | 12.15306 |
| Macrophages M2 | 5.550963 | 10.91598 | 6.501628 | 2.58123 | 3.564988 | 3.176274 | 0.832522 | 7.584844 | 3.754934 | 23.91774 |
| Dendritic cells resting | 0.238665 | 0.181611 | 0.170402 | 0.066802 | 0.322061 | 0.155723 | 0.054476 | 0.208467 | 0.046268 | 0.295685 |
| Dendritic cells activated | 0.066458 | 0.225106 | 0.015984 | 0.067538 | 0.236003 | 0.252429 | 0.006338 | 0.914938 | 0.15047 | 2.544651 |
| Mast cells resting | 6.412128 | 7.80389 | 0.412265 | 0.859021 | 2.298151 | 0.812086 | 0.786971 | 6.529429 | 2.063162 | 8.096318 |
| Mast cells activated | 0.364044 | 2.244389 | 2.831652 | 0.533947 | 0.694725 | 0.478199 | 0.046016 | 2.622022 | 0.106199 | 0.402131 |
| Eosinophils | 0.080789 | 0.301347 | 2.053105 | 0.7589 | 0.168208 | 0.773169 | 0.01932 | 0.119216 | 0.129837 | 0.498733 |
| Neutrophils | 0.642991 | 3.607262 | 0.833096 | 0.754131 | 0.19815 | 0.480001 | 0.367285 | 5.575776 | 0.419926 | 2.55837 |
| Abbreviations: NK, natural killer | | | | | | | | | | |
