# Supplementary material for: Immune-related pan-cancer gene expression signatures of patient survival revealed by NanoString-based analyses
Source: PLoS One. 2023 Jan 17;18(1):e0280364. doi: 10.1371/journal.pone.0280364 (PMC9844904; doi:10.1371/journal.pone.0280364)
Supplement: Attachment — Submitted filename: Alberto De Angelo et al Comments.docx [file pone.0280364.s011.docx]

The research article titled “Immune-related pan-cancer gene expression signatures of patient survival revealed by NanoString-based analyses.”, by D’Angelo et al, describes about the gene expression anlysis in multiple cancers to identify the novel immune related gene signature tht could be potentially used in cancer prognosis and therapy response analysis. Overall, the manuscript is nicely written, and methods used to generate the data is robust and machine based. Authors have highlighted the novel gene signature associated with solid tumors of patients with shorter survival. Results and discussion are appropriately presented in the manusctipt. Following are the minor comments for authors that need be addressed before considering the manuscript for the publication in the journal PLOS One. Authors are suggested to revise the manuscript to address the following comments:

1. Line 53-56: Please change the sentence as “A better understanding of transcriptional changes in immune cell related genes associated with cancer progression, and their significance in disease prognosis is therefore needed.”
2. Line 62-63: Change the sentence as “Most of the genes identified have previously been reported as relevant in one or more cancer types.”
3. Line 79-82: Sentence is complex and can be simplified as “Such variable success is probably linked to the complexity of the tumor microenvironment, which involves cell-cell interactions among multiple cell types, with accompanying dynamic genomic and epigenetic characteristics (7–9).
4. Section 3.2: Authors are suggested to explain the significance of clustering in the result section. It will help in understanding the data more clearly.
5. Overall, figure legends are too short and not found sufficient to explain the figures. For example, in figure legend 3, it is desired from authors to elaborate the figure legend, mentioning the statistical analysis in the figure a, and mention the details for the color scale of heatmap. In total, all figure legends need more description for better understanding, including the descriptions of X and Y axis.
6. As GAGE1, MAGEC2 and SSX1, is novel finding as a cluster for solid malignancies, authors are suggested to include more description for these genes and how these can be suitable for disease prognosis.
7. Figure 6-8: legends need to be more descriptive of figure and require a rewrite as they look like description of result and methods, rather than a description of figure.
8. Line 321-327: Please describe what does it means to have high or low immune cell types in solid tumors, in context of survival and treatment response.
9. Line 328-340: Immune cell analysis of long and short surviving patients have been compared by the authors, comparing different cancers. However, in a single cancer type, long and short survival patients have not been compared in a single cancer type. Authors are suggested to include that analysis to validate the gene signature, reported by the authors.
10. In figure 9, Y axis detains are missing. Authors are advised to add the details.
